# Digital doppelgangers in psychiatry

**DOI:** 10.1007/s44192-026-00517-1

**Published:** 2026-07-23

**Authors:** Khaled Elbarbary, Sheikh Shoib

**Affiliations:** 1 Department of medicine, Mansoura University, El-dakahlia, Egypt; 2 Department of health services, Srinagar, Kashmir India

**Keywords:** Digital phenotyping, Precision psychiatry, Artificial intelligence, Mental health surveillance ethics, Behavioral digital biomarkers

## Abstract

Digital doppelgangers are individualized, continuously updated digital representations of a person constructed from behavioral, physiological, and contextual data streams, including smartphone metadata, wearable sensor outputs, social media activity, and environmental sensors. Whereas conventional digital twins in physical medicine primarily replicate anatomical structures and physiological parameters, psychiatric digital doppelgangers are designed to capture dynamic mental states, emotional trajectories, and behavioral pathways through aggregated multimodal digital traces. Preliminary research suggests potential clinical utility across several domains, including earlier detection of depressive and bipolar episodes, risk stratification for suicidal crises, and individualized treatment planning; however, most applications remain at the feasibility and proof-of-concept stage and have not yet achieved prospective clinical validation. Implementation raises substantive challenges, including informed-consent complexity under fluctuating decisional capacity, algorithmic bias arising from non-representative training datasets, diagnostic ambiguity in the interpretation of behavioral signals, inequitable access to required technology infrastructure, and the risk of reconfiguring the therapeutic relationship into a surveillance mechanism. Responsible development requires interdisciplinary collaboration among clinicians, technologists, ethicists, regulators, and patient communities, alongside robust ethical frameworks, prospective validation regimes, and genuine patient partnership throughout the development cycle. Digital doppelgangers represent a conceptually distinct but adjacent framework to digital twins, digital phenotyping, and AI-driven cognitive science models; their trajectory in psychiatry depends on whether technological ambition is matched by equally rigorous governance and a primary commitment to patient welfare.

## Introduction

The emergence of digital doppelgangers, broadly defined as individualized, continuously updated digital representations of a person constructed from behavioral, physiological, and contextual data, marks a potential shift in how psychiatric assessment could be conceptualized. In contrast to digital twins in physical medicine, which primarily replicate anatomical structures and organ-level physiological processes for surgical simulation or disease modeling, psychiatric digital doppelgangers aim to capture the dynamic nature of mental states, emotional patterns, and behavioral pathways through aggregated multimodal digital traces [1]. They also differ from digital phenotyping, which refers to the moment-by-moment quantification of the individual-level human phenotype using data from personal digital devices and is primarily a passive measurement construct rather than a predictive simulation framework [2, 3]. Unlike AI-driven cognitive science models, which use computational and machine-learning architectures primarily as simulators or estimators of latent cognitive processes at the theoretical level, digital doppelgangers are oriented toward integrating heterogeneous real-world data streams into a persistent, updatable individual representation intended for direct clinical use [4].

Wang draw a relevant distinction between AI models, which map data to predictions or latent dynamics; AI systems, which embed such models within data pipelines, interfaces, and governance structures; and AI agents, which are goal-directed and capable of planning and tool use. A digital doppelganger most closely corresponds to an AI system in this taxonomy: its clinical value depends not on the predictive model alone but on the surrounding infrastructure of data governance, clinician interface, and oversight protocols. The conceptual positioning of digital doppelgangers relative to adjacent constructs is further clarified in Table 1.

**Table 1:**
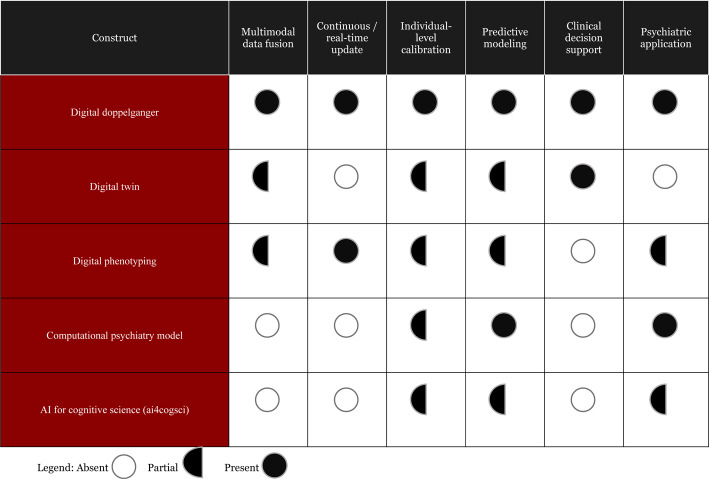
Feature matrix comparing digital health constructs

This article addresses the potential benefits and risks of applying this construct in clinical psychiatry, acknowledging that the field is at an early stage where feasibility studies predominate and prospective clinical validation remains limited.

### Current state of digital mental health monitoring

Present-day research regarding the use of digital technologies for psychiatric assessment focuses on individual data modalities rather than integrated digital doppelgangers. Researchers are assembling behavioral digital biomarkers from smartphone usage patterns, wearable device sensor outputs, social media content, and linguistic data in preliminary attempts to estimate mental states, and early feasibility evidence supports the potential of this approach [2, 3, 5]. For instance, evidence from systematic review suggests that smartphone metadata and wearable accelerometry data show associations with depressive and bipolar episode markers in feasibility cohorts, though individual-level predictive accuracy at clinically actionable thresholds has not yet been established in prospective, adequately powered trials [5, 6].

Wang describe how AI in psychiatry currently functions as an estimator within digital phenotyping pipelines that integrate smartphones, wearables, and web browsing history to predict near-term mood shifts, relapse risk, and treatment response, with observable inputs including sleep regularity, heart-rate variability, step counts, GPS entropy reflecting mobility diversity, call and text rhythms, and speech features such as pause rate and prosody. Large-language-model-based early-warning schemes can fuse daily speech snippets and activity traces, embed acoustic, textual, and behavioral features, estimate movement between symptom-network states such as sleep disruption transitioning to low mood, and trigger clinician or patient feedback when the probability of a critical transition rises [4]. Temporal-network and dynamical-systems approaches, which Wang situate within a broader complex-systems paradigm for psychiatry, recast disorders as evolving configurations of mutually reinforcing symptoms exhibiting attractor states, bifurcations, and critical transitions; hour-scale fluctuations in suicidal ideation have been documented, underscoring the need for time-sensitive, dynamic risk models rather than static assessments.

A trimodal framework integrating ecological momentary assessment, physiological biosensor measurements, and speech emotion recognition illustrates the methodological direction toward multimodal, longitudinally sensitive mental health monitoring [7]. Practical implementations of this multimodal approach include systems that combine structured cognitive architectures with machine learning models trained on ecological momentary assessment data to simultaneously detect and forecast stress, anxiety, and depression symptoms: one such system, using a cognitive architecture that simulates Theory of Mind, achieved 7-day forecasting accuracies of up to 87.68% from text data and outperformed a widely used conversational agent in reducing self-reported stress and anxiety levels in a controlled empirical study [8]. AI-driven psychotherapy systems and conversational agents have also begun to incorporate data from such streams to support personalized intervention, though interpretability, user engagement, and clinical integration remain active challenges [9, 10].

None of these tools has progressed substantially beyond the research prototype stage in terms of validated, routine clinical implementation. Where the concept of digital doppelgangers diverges from these preceding tools is in their proposed integration of multiple heterogeneous data streams into a persistent, continuously updated individual model intended not only to describe current states but also to generate predictions of future psychiatric states based on an individual-specific behavioral phenotype [3]. This shift from passive data collection to active, individualized simulation represents a qualitative advance in ambition relative to current digital phenotyping; whether the necessary predictive validity can be achieved at the individual level and within clinically acceptable error bounds remains to be established.

## Redefining psychiatric assessment and monitoring

Digital doppelgangers could, in principle, extend psychiatric assessment beyond intermittent clinical encounters. By integrating smartphone usage data, wearable physiological outputs, social media activity, and environmental sensor signals, such models might achieve higher temporal resolution in tracking mood fluctuations, sleep patterns, social interaction frequency, and behavioral changes than is possible within scheduled appointments. Whether this granularity translates into earlier and more reliable detection of psychiatric disorder onset or relapse remains a research question: preliminary studies suggest that subtle changes in digital behavioral patterns may precede clinically apparent symptom emergence by days to weeks in some cohorts, but these findings derive largely from small, selected research populations and have not been validated in diverse, community-based psychiatric samples [5, 6].

Real-time risk stratification is among the most frequently cited potential applications. Traditional suicide risk assessment relies primarily on patient self-report and clinician judgment during brief encounters, a limitation that digital monitoring might partially address [6]. Digital doppelganger systems could hypothetically identify multivariable signal patterns associated with escalating risk, such as sustained reductions in social media engagement, sleep-wake cycle fragmentation, mobility restriction, or shifts in linguistic valence, and alert clinicians to emerging concern before a crisis threshold is reached [6, 11]. The temporal dynamics of suicidal ideation are particularly relevant here: documented hour-scale fluctuations in suicidal ideation underscore the limitations of episodic assessment and the theoretical advantage of continuous monitoring for time-sensitive risk stratification [4]. It is critical, however, to distinguish between group-level risk associations and individual-level predictive accuracy: the former is increasingly supported by preliminary evidence, whereas the latter has not been demonstrated at the specificity and sensitivity levels required for safe clinical deployment. False-positive alerts carry real harms, including unnecessary clinical escalation, patient anxiety, and erosion of trust; false negatives carry the risk of missed intervention. Any clinical deployment pathway must therefore specify acceptable thresholds for both error types, alongside clinician review protocols, audit trails, and mechanisms to manage alert fatigue [11].

A credible path to clinical utility would require, at minimum, prospective validation in real-world psychiatric populations across diagnostic categories; pre-specified, clinically meaningful target outcomes such as reduced hospitalization rates or earlier treatment initiation; transparent reporting of sensitivity, specificity, positive predictive value, and negative predictive value rather than generic accuracy metrics; and systematic procedures for clinician review of algorithmic outputs, including escalation protocols.

Similarly, early warning systems for psychotic or manic episodes could be developed by monitoring deviations from individually calibrated behavioral baselines, though the available evidence base remains limited to small feasibility studies and has not established prospective clinical validity [5].

**Fig. 1:**
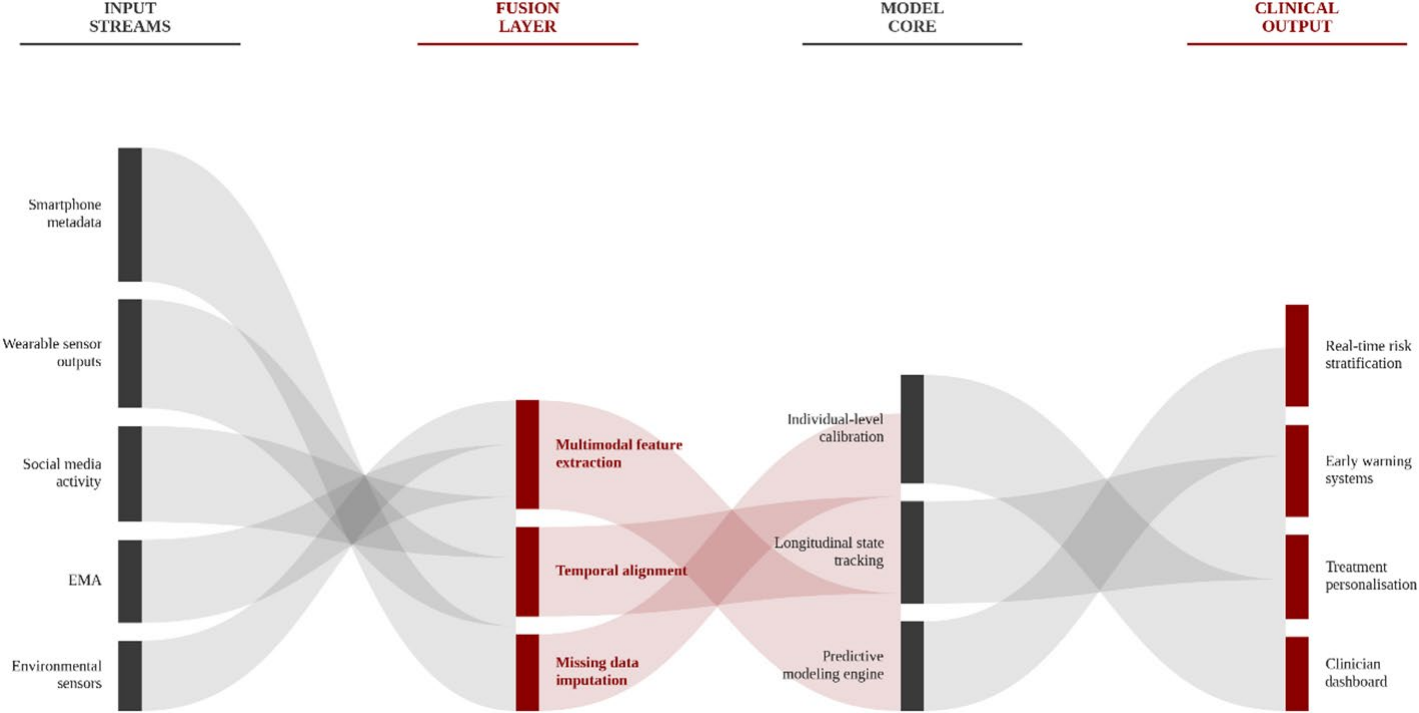
Multimodal data integration architecture of a digital doppelganger

Interpretation of digital behavioral signals requires explicit acknowledgment of confounders. Changes in social media posting frequency, mobility patterns, or sleep timing may reflect occupational schedules, shift work, international travel, deliberate digital detox, cultural or religious observances, shared device use, or socioeconomic constraints rather than psychiatric state changes [12]. Practical safeguards should include structured mechanisms for patients to provide contextual tags, uncertainty quantification in model outputs, regular calibration against clinical outcomes, and mandatory clinician review before any action is taken on algorithmic flags. Wang recommend site-specific cross-validation and local recalibration when models are deployed across different clinical populations or settings, a requirement directly applicable to digital doppelganger systems moving from development cohorts to real-world practice. The architecture underlying this integration, spanning five input streams, a multimodal fusion and individual calibration core, and four clinical output categories, is depicted schematically in Fig. 1.

### Personalization versus standardization

The prospect of individualized mental health treatment stems from the theoretical ability of digital doppelgangers to detect individual-level patterns of treatment response, environmental reactivity, and behavioral trajectory. Rather than applying population-derived treatment algorithms uniformly, clinicians could in principle tailor interventions to an individual’s unique digital phenotype, potentially improving treatment timing and modality selection beyond current clinical algorithms [3]. Intelligent cognitive assistants reviewed by Kolenik and Gams illustrate the technical landscape for personalization; the most effective systems combined comprehensive user models, classification-based assessment of mental states, and individually tailored behavioral interventions delivered through natural language dialogue, a technical architecture that shares substantial conceptual overlap with the personalization logic a digital doppelganger would require [13]. At a more advanced level, Kolenik developed a computational psychotherapy system that trained longitudinal machine learning models on idiographic ecological momentary assessment data, enabling personalized intervention selection driven by individually calibrated symptom profiles, thereby demonstrating the practical feasibility of idiographic modeling tied to personalized behavioral change support. AI-driven systems have begun to demonstrate personalization capabilities in controlled settings, and early work on AI-based psychotherapy platforms has shown the ability to adapt interaction content to inferred user states, though the robustness and generalizability of this personalization across diverse clinical populations require further study [10, 14].

Despite the apparent individuation of digital doppelgangers, they are not constructed independently of population-level data. The underlying algorithms require training on population datasets to establish baseline parameters, after which individualization proceeds through iterative updating with person-specific data. This dual-level methodology implies that populations underrepresented in training data, including cultural minorities, people with limited technology access, neurodivergent individuals, and people in low- and middle-income settings, may experience systematically less accurate models [12, 15].

**Fig. 2:**
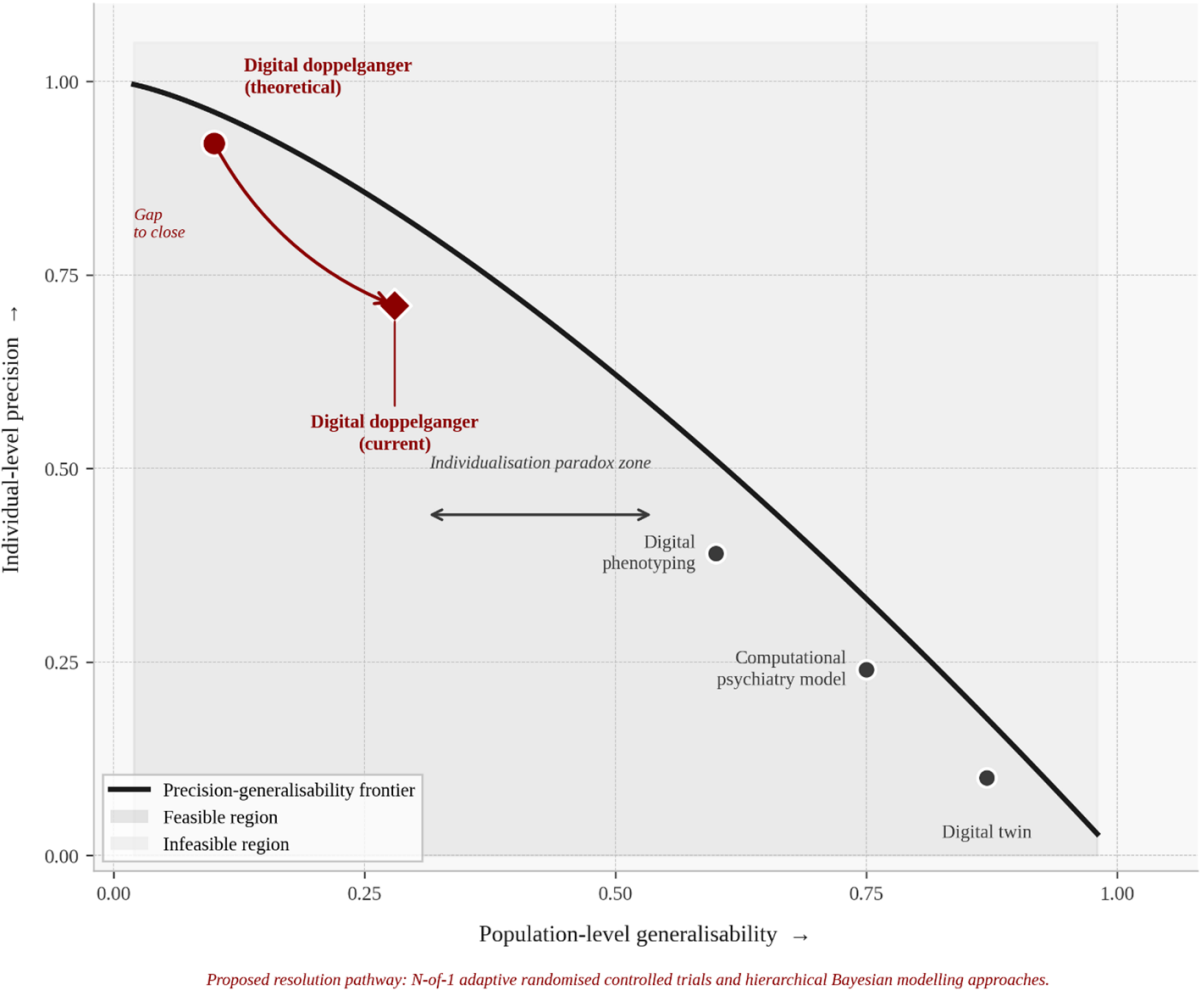
Conceptual precision-generalisability frontier illustrating the individualisation paradox

This individualization paradox creates tension with the standardization that evidence-based practice requires. When each patient’s digital representation yields unique model outputs, establishing the scientific integrity of treatment recommendations derived from those outputs becomes methodologically demanding. Aligning the precision psychiatry paradigm with existing diagnostic frameworks such as DSM-5-TR and ICD-11 raises open questions: digital behavioral signals do not map directly onto categorical diagnostic criteria, and their integration into formal diagnostic reasoning will require both regulatory guidance and clinical consensus on interpretation standards. This tension is conceptualised in Fig. 2.

### The therapeutic relationship in the digital era

The integration of digital doppelganger systems into clinical care could substantially alter the therapeutic relationship in both directions. On the positive side, access to between-session behavioral and physiological data could enhance clinicians’ understanding of a patient’s lived experience, potentially deepening therapeutic engagement and enabling more timely and contextually informed treatment adjustments. Quantitative signals about sleep architecture, physical activity, and social interaction patterns could complement patient self-report and support the working alliance by giving both clinician and patient a shared observational basis for collaborative reflection [1].

**Fig. 3:**
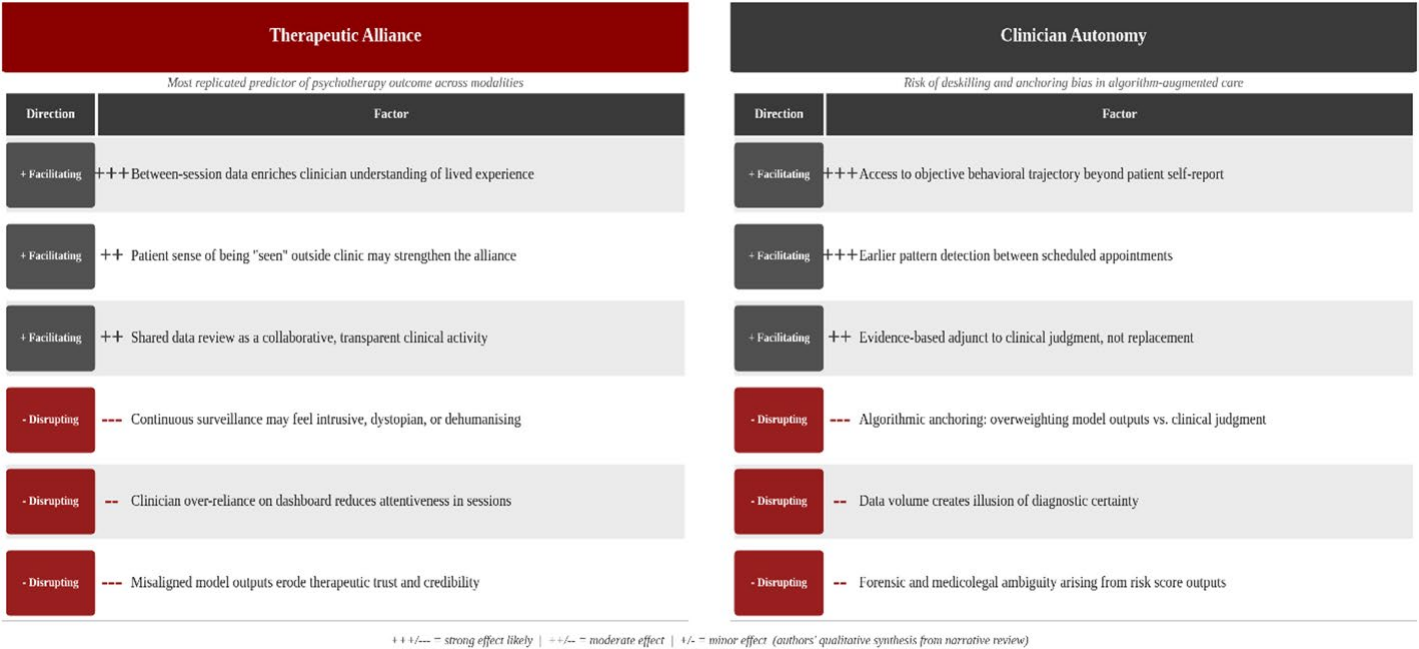
Bidirectional effects of digital doppelganger integration on therapeutic alliance and clinician autonomy

The concept of therapeutic alliance, which is among the most replicated predictors of psychotherapy outcome across modalities, is particularly relevant here: digital doppelganger systems could either strengthen alliance by giving patients a sense that their between-session experience is seen and valued, or undermine it by introducing a surveillance dynamic that reduces the relational safety required for genuine disclosure [1]. Continuous monitoring of digital behavior for clinical signals risks transforming the therapeutic relationship into a surveillance regime. The awareness that one’s digital activity is being analyzed for psychiatric signs may induce performative behavior, self-censorship of online activity, or deliberate alteration of digital footprints, paradoxically degrading the authenticity and ecological validity of the data on which the model depends. This behavioral adaptation could cause the digital doppelganger to diverge from the patient’s actual psychiatric state over time, reducing both model accuracy and clinical utility [3]. The principal facilitating and disrupting factors for both therapeutic alliance and clinician autonomy are presented in Fig. 3.

An alternative trajectory is conceivable; if a well-calibrated model achieves sufficient predictive validity from periodic rather than continuous sampling, the scope of continuous monitoring could be reduced, diminishing privacy intrusion while maintaining clinical utility. Whether periodic update cadence is sufficient for clinically meaningful prediction is itself an empirical question that requires prospective testing. The design of digital doppelganger interfaces, specifically the extent to which patients can actively engage with their own model outputs, modify contextual inputs, and exercise control over data visibility, will substantially influence whether these systems are experienced as empowering or intrusive. Engagement and interaction design principles identified in the review by Kolenik and Gams, including the importance of natural language dialogue, adaptive response generation, and context-sensitive strategy selection, are directly applicable to the interface design requirements of digital doppelganger systems. User experience research has identified significant variation in patient perceptions of AI-mediated therapeutic tools and should inform interface design from the earliest development stages [14].

### Interpretive challenges and diagnostic ambiguity

The translation of digital behavioral patterns into clinically actionable knowledge involves substantive interpretive challenges. Digital monitoring systems excel at identifying statistical associations and temporal patterns but cannot by themselves establish causal mechanisms or contextual meaning. Reduced social media engagement, for instance, may reflect a depressive episode, a deliberate digital detox, occupational demands, a change in social network composition, travel, cultural observance, or, in the case of shared devices, another household member’s usage pattern [12]. The risk of misinterpretation is compounded when algorithms lack structured access to a patient’s life context.

The volume and apparent precision of continuous data can create an illusion of diagnostic certainty. When a digital doppelganger model generates a high-confidence risk alert, clinicians may experience algorithmic anchoring, a tendency to overweight algorithmic output relative to clinical judgment and the patient’s own account. Wang recommend that each prediction or recommendation should include a brief rationale tied to concrete evidence, such as specific symptom trajectories, with escalation workflows for ambiguous cases and that model patterns should be linked back to cognitive and neuroscientific theory to move from correlations to plausible mechanisms. When behavioral indicators conflict within the same data stream or across modalities, the procedures for resolving such conflicts must be explicitly defined in clinical protocols rather than left to ad hoc clinician discretion.

The relationship between digital behavioral signals and established diagnostic frameworks such as DSM-5-TR and ICD-11 requires explicit conceptualization. Digital doppelganger outputs do not map directly onto categorical diagnostic criteria, and the field has not established how such outputs should inform, modify, or potentially conflict with formal diagnostic reasoning. Addressing this gap is necessary before clinical deployment and will require both regulatory guidance and clinical consensus on interpretation standards.

### Ethical concerns and privacy paradoxes

The application of digital doppelgangers in psychiatry raises ethical concerns that extend beyond standard medical privacy considerations [11, 16]. Structuring these concerns within the three principles of biomedical ethics; autonomy, beneficence and non-maleficence, and justice, clarifies both the nature of the risks and the requirements for mitigation [17]. A cross-construct comparison of concern levels across all three principles, with sub-domain specificity, is presented in Table 2.

**Table 2:**
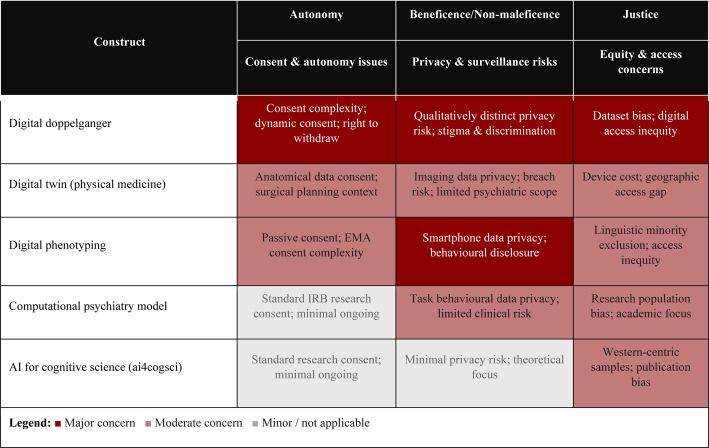
Ethics concern levels across digital health constructs

1. Autonomy

Informed consent is particularly complex in psychiatric contexts: patients may not be able to fully anticipate how their behavioral data will be analyzed, what inferences will be drawn, or how model outputs will influence clinical decisions [11]. The fluctuating nature of mental illness, including episodes of diminished decisional capacity, can coincide with the periods when continuous digital monitoring is most clinically relevant, creating situations in which standard capacity-based consent procedures may be inadequate. Standard consent procedures may need to be supplemented by advance directives specifying a patient’s preferences for data use during periods of impaired capacity, and by ongoing, granular consent processes that allow modification or withdrawal at any time [18].

2. Beneficence and non-maleficence.

The behavioral comprehensiveness of digital doppelganger data, encompassing mobility, communication patterns, biometrics, and social interactions, creates privacy exposure that is qualitatively different from standard medical records. Mental health data carries stigma and discrimination risks, making unauthorized disclosure, re-identification from supposedly anonymized behavioral data, or secondary use of data particularly harmful [11, 16]. Technical mitigations that should be considered from the earliest development stages include on-device processing to minimize data transmission, federated learning to train models without centralizing raw personal data, differential privacy to protect against re-identification, strict data minimization to collect only variables with demonstrated clinical relevance, defined data retention limits, and access controls with auditability requirements [11]. Wang further specify that governance frameworks must distribute control through data trusts, align system design with stakeholder values through participatory co-design, and make model performance auditable through model cards and bias audits, with Finland’s MyData initiative cited as an operational template for granular individual consent.

3. Justice.

The potential for predictive insight into future psychiatric states raises concerns analogous to genetic discrimination, specifically the possibility that algorithmic risk scores could be used by insurers, employers, or legal systems in ways that violate individuals’ right to an open future [16]. Responsible AI integration in mental health research therefore requires clear regulatory boundaries on secondary data use, transparency in algorithmic decision-making, and explicit accountability mechanisms for adverse outcomes [17].

### Algorithmic bias and health equity

Digital doppelgangers may reinforce and magnify pre-existing biases in psychiatric diagnosis and treatment. Training datasets for these systems tend to overrepresent the digital behaviors of individuals with greater access to and habitual use of technology, generating models that perform less accurately when applied to populations from lower-resource settings, older age groups, or different cultural and linguistic backgrounds [12, 15]. Cultural variation in digital communication norms, emotional expression styles, and technology use patterns may be systematically misclassified as pathological by algorithms calibrated on narrow demographic samples. Wang document that biased error rates for underrepresented groups, including under-detection of depression as a named example, represent a fundamental challenge for AI systems in psychiatry, and that equity must be addressed through subgroup performance auditing and fairness-oriented training rather than treated as an afterthought.

The risk of over-pathologizing normative behavioral variation is greatest for groups whose digital behavior deviates from algorithmic training norms for reasons unrelated to psychiatric state. Neurodivergent individuals, cultural and linguistic minorities, people in shift-working or non-standard occupational settings, and people in low- and middle-income settings may receive algorithmically elevated risk scores that reflect deviation from a normative baseline rather than genuine clinical deterioration [12, 15]. A related structural concern is access inequity: digital doppelganger systems require smartphone ownership, reliable internet connectivity, and compatible wearable devices, and if these tools are developed and deployed primarily for well-resourced populations, they risk widening the already substantial gap in mental health service access rather than narrowing it [11, 19]. Kolenik and Gams specifically identify group exclusion, encompassing older adults, people from the lowest socioeconomic class, and culturally specific groups, as a primary equity risk of persuasive digital mental health technologies, and argue that such technology should function as a complement to, rather than a replacement for, professional mental healthcare, specifically to avoid exacerbating existing inequalities in access [19].

**Fig. 4:**
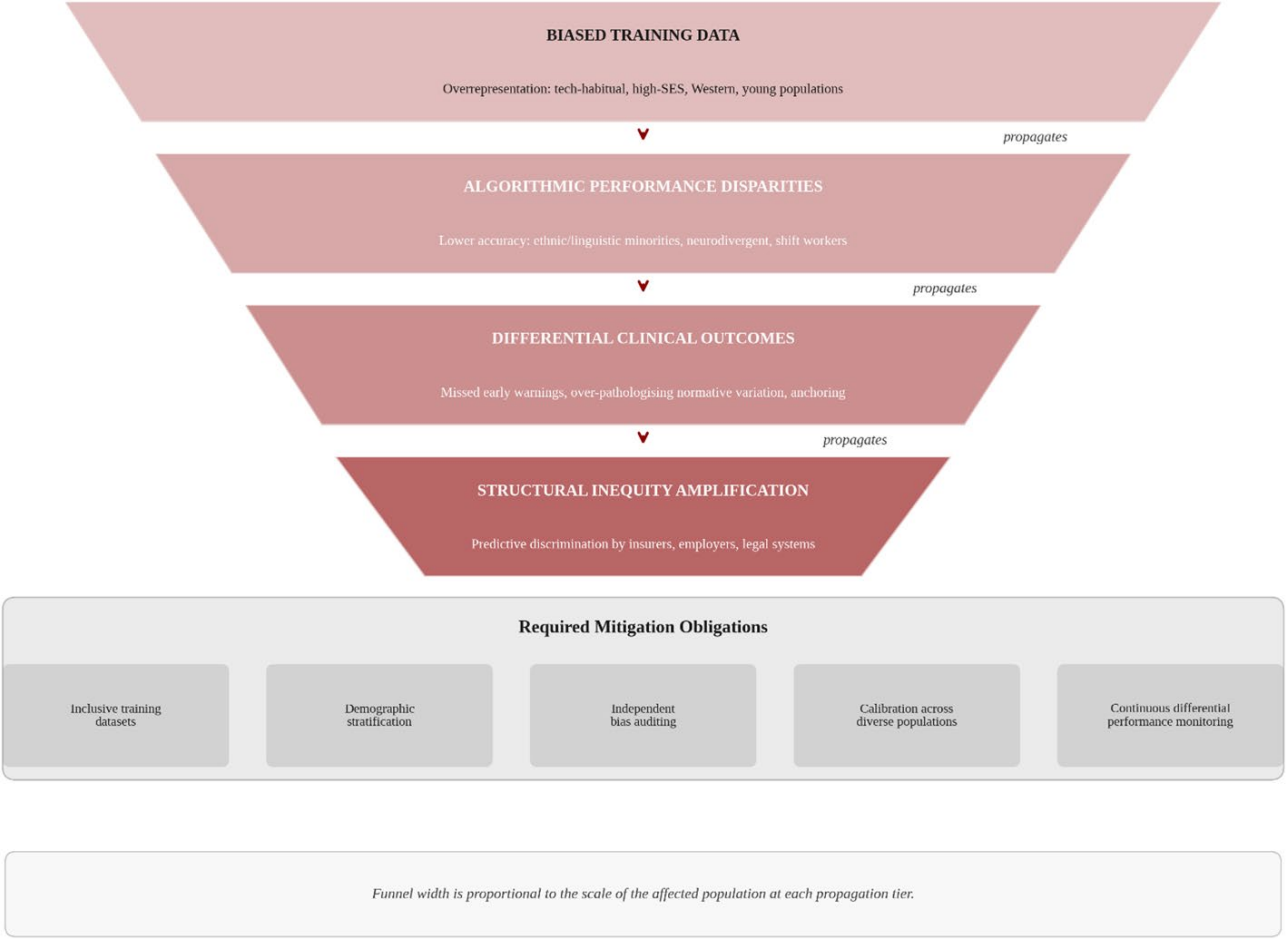
Cascade mechanism of algorithmic bias and health equity implications

These equity concerns imply a set of development obligations: inclusive training datasets with demographic stratification and fairness auditing, independent bias evaluation before deployment, calibration studies across diverse populations, and explicit monitoring of differential model performance across demographic groups in post-market surveillance. Participatory design approaches that meaningfully involve underrepresented patient communities in the development and governance of these systems are an additional, underdeveloped area where progress is needed [4, 17]. This propagation follows a four-tier cascade from biased training data through performance disparities and differential clinical outcomes to structural inequity amplification, as illustrated in Fig. 4.

### Responsible development and future directions

Successful integration of digital doppelgangers into clinical practice would require substantial technological and organizational infrastructure investment, including electronic health record architectures capable of ingesting and displaying longitudinal multimodal behavioral data streams, clinician training in data interpretation, and institutional governance frameworks that define accountability for model outputs [11, 17]. Current electronic health record systems are not designed for this purpose, and workflow integration will require prospective design rather than post hoc adaptation.

From a forensic perspective, the clinical and legal implications of digital doppelganger outputs require explicit pre-specification. The use of algorithmic risk scores in involuntary treatment decisions, risk-based hospitalization, or forensic risk assessment raises questions of due process, evidentiary standard, and liability that extend beyond existing regulatory frameworks for clinical decision support software. The potential for misuse of persistent behavioral profiles, including in insurance underwriting, employment contexts, or coercive monitoring by family members or legal guardians, is a concrete risk that governance frameworks must explicitly address rather than treat as a theoretical concern.

Responsible development of digital doppelgangers in psychiatry requires unprecedented interdisciplinary collaboration among technologists, clinicians, ethicists, regulators, and patient communities [11, 17]). Technical innovation must proceed alongside durable ethical frameworks, regulatory protocols, and evidence-based validation regimes, including prospective trials that specify target outcomes, acceptable error rates, and procedures for managing algorithmic failures. The Kolenik study provides a replicable model for this validation approach [8]. The system was tested in a controlled empirical study of 42 participants, achieving statistically significant reductions in self-reported stress (*p* = 0.004) and anxiety (*p* = 0.008). This dual-evaluation model, computational benchmarking followed by controlled clinical testing, represents the minimum evidentiary standard that digital doppelganger systems should be expected to meet before deployment. Wang propose several development priorities directly applicable to this context: establishing robust methodologies for validating dynamic models against long-term human data across diverse contexts; building trustworthy systems through participatory ethical frameworks, algorithmic fairness auditing, and transparent human oversight mechanisms; and enabling sustainable deployment via low-resource AI solutions and effective augmentation models that support clinicians rather than replace them. Kolenik and Gams further stress that genuine societal and patient partnership is required throughout development to ensure that the benefits of such technology are realized without deepening existing inequalities [19].

**Fig. 5:**
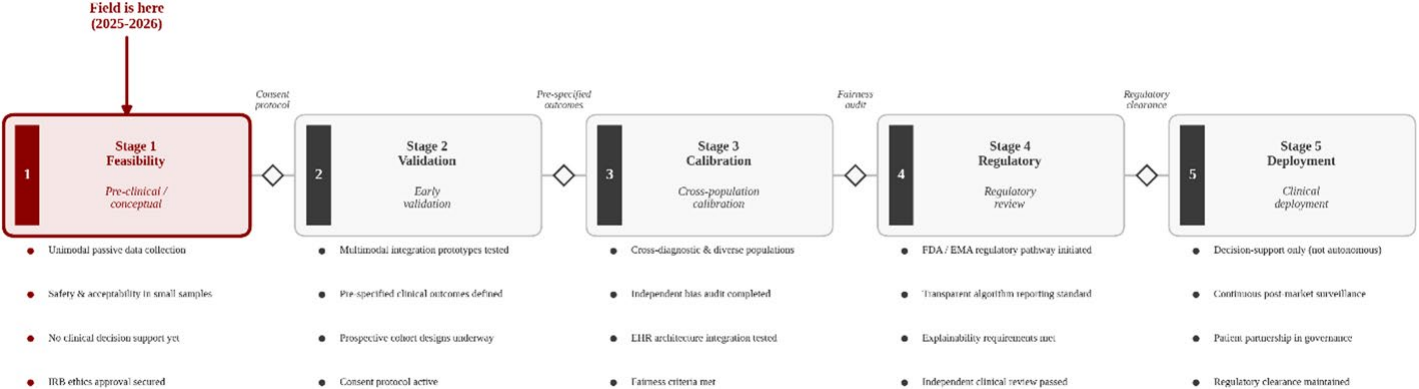
Proposed staged clinical translation pathway

Rather than pursuing wholesale adoption, the field requires staged, evidence-driven progression with robust governance, continuous evaluation, and genuine patient partnership throughout the development cycle. The objective should be to position digital doppelgangers as decision-support tools that augment rather than replace clinical judgment, with the human dimensions of the therapeutic relationship preserved as a non-negotiable constraint on system design [9, 10]. A proposed five-stage pathway with mandatory evidence-based gates, current field position, and a continuous governance obligation is presented in Fig. 5.

## Conclusion

Digital doppelgangers represent a conceptually distinct and potentially significant development in precision psychiatry, differentiable from digital phenotyping, digital twins in physical medicine, and AI-driven cognitive science models by their integration of multimodal behavioral data into persistent, updatable individual representations intended for direct clinical use. Current evidence supports the feasibility of individual data modalities within this framework but does not yet establish the prospective clinical validity required for deployment. Responsible progress requires rigorous prospective validation, structured ethical governance addressing autonomy, equity, privacy, and potential for misuse, and a development paradigm that positions patient welfare above technological advancement. The fundamental question is whether the transformation digital doppelgangers could bring to psychiatric practice will ultimately enhance human flourishing or introduce new mechanisms of surveillance and inequity, and the answer will depend as much on governance and values as on technology.

## Data Availability

No datasets were generated or analysed during the current study.
